# “I got to catch my own baby”: a qualitative study of out of hospital birth

**DOI:** 10.1186/s12978-022-01355-4

**Published:** 2022-02-14

**Authors:** Mickey Sperlich, Cynthia Gabriel

**Affiliations:** 1 University at Buffalo School of Social Work, 685 Baldy Hall, Buffalo, NY 14260 USA; 2 University of Michigan Women’s Studies, 204 S. State St, Ann Arbor, MI 48109 USA

**Keywords:** Out of hospital birth, Community birth, Unassisted birth, Freebirth, Respectful birth, Traumatic birth, Sexual abuse survivors, Health disparities, Black maternal health

## Abstract

**Background:**

About 1.6% of planned births in the United States occur out of hospitals. Studies indicate that planned out-of-hospital birth (OOHB) is safe and satisfying for women; however, there is great variation among ethnic groups, and Black women are underrepresented. A recent phenomenon is the choice to have an unassisted birth (UAB) with no midwife or other professional maternity care attendant. The purpose of this study is to fill a gap in understanding reasons for choosing OOHB or UAB for two clinically important sub-groups of women: Black women, and women who have experienced childhood physical or sexual abuse.

**Methods:**

This study recruited 18 women who had an OOHB or UAB and who identified as either Black or survivors of trauma to participate in in-depth qualitative interviews concerning their choice to give birth out of hospital. A grounded theory approach was utilized that involved a discursive process of data collection, coding textual passages to identify focused themes, memo writing to document analytic decision-making, and eventual conceptual modeling.

**Results:**

All 18 participants endorsed a history of trauma. Focused coding to identify inherent concepts led to the emergence of a theoretical model of the arc of decision-making around choice of place of birth and birth attendant, or lack thereof. Women may choose OOHB or UAB because of a previous trauma, or because they feel discriminated against by healthcare professionals, either because of skin color, age, pregnancy, weight, or some other health condition. Women may choose OOHB or UAB because it affords more control during the process of giving birth.

**Conclusion:**

Previous trauma and experiences of discrimination were influential factors for women in the study sample in their choice of birthplace setting and choice of provider. These findings can inform clinical understanding for birth professionals, including doctors, midwives, doulas, nurses, social workers, and psychologists, and contributes more broadly to the national conversation about birth choices in the USA.

## Plain English Summary

This study shares information from qualitative interviews with Black women and women who are survivors of abuse regarding their choice to have a planned out-of-hospital birth, or to choose an unassisted birth (UAB) with no midwife or other professional birth attendant. Black women are less well represented among those who choose OOHB, and little is known about the reasons that they may choose OOHB. Previous studies show that women who have experienced childhood physical or sexual abuse may prioritize having a sense of control and autonomy during their birthing experiences; however, little is known about their specific choice for OOHB. Our study recruited 18 women who had an OOHB or UAB and who identified as either Black and/or survivors of trauma to participate in in-depth interviews concerning their choice to give birth out of hospital. Through qualitative research methods, we analyzed transcripts of these interviews and developed a theoretical model about women’s decision making related to OOHB or UAB. We found that women may choose OOHB or UAB because of a previous trauma, or because they feel discriminated against by healthcare professionals, either because of skin color, age, pregnancy, weight, or some other health condition, and that choosing OOHB or UAB allowed them to have more control during the process of giving birth. Understanding the role that previous trauma and experiences of discrimination play in birthplace choice may help birth professionals to consider bodily autonomy, physical and emotional safety, anti-racism, and independence as important factors in their clinical interactions with birthing women.

## Introduction

About 1.6% of births in the United States are “community births” that occur out of hospitals, including births in a residence (i.e., home births), freestanding birth center, clinic, doctor’s office, or other location [1]. A tiny percentage of these are planned unassisted births. Why women choose community birth has been somewhat studied; however, most women included in such studies have been highly educated, married, and white [2]. More unassisted births (UABs) occur to Black women than white women [3], but there are no published qualitative studies of out of hospital birth (OOHB) or UAB that include Black women’s voices. Previous studies indicate that women may choose OOHB for a sense of control, especially after experiencing previous trauma [4]. The present study aims to investigate the OOHB decision-making of two clinically important and understudied subgroups of women: Black women and women who have experienced childhood trauma.

### Choosing community birth: black women and women survivors of childhood abuse

The experiences of Black women are noticeably absent from the literature about community birth. One of the few qualitative studies considering why women choose attended OOHB included a participant sample which was 87% white [5]. Studies on planned community birth in the Netherlands and Canada do not discuss ethnicity-related disparities in experience or access [6, 7]. An integrative review of twenty-one research-based papers examined reasons that women choose place of birth, but never touched on the relevance of personal demographic factors [8]. How racial identity and experiences of discrimination influence women’s decision-making about place of childbirth has not been addressed.

Also missing are the voices of women survivors of abuse. Stress has long been known to adversely affect the physiological processes of labor [9]. Women who are of a racialized minority may experience greater stress in the hospital setting. It is not known if they might have better psychological and/or physiologic outcomes if out-of-hospital models of care were more widely available in the United States, as they are in many nations with better maternity outcomes (e.g., Canada, Northern Europe). Quantitative data indicates that women with previous trauma have worse perinatal outcomes, and that stress system dysregulation may mediate these adverse outcomes [10–12]. Systemic racism and discrimination have been studied as unavoidable traumas for people of color in the United States with known impacts on health outcomes, including disproportionate risk for posttraumatic stress disorder (PTSD) in pregnancy [13], and adverse birth outcomes [14]. As such, highlighting the reasons that trauma survivors and Black women choose community birth is important.

### Unattended OOHB and UAB

Although planned OOHB has been shown to be safe in multiple studies, especially when guidelines for hospital transfer are well-developed [15, 16], OOHB has generated much controversy. One reason is that some authors cite birth certificate data that do not differentiate between planned or unplanned OOHB. Studies that do differentiate between planned and unplanned out-of-hospital births reveal that unplanned out-of-hospital births are known to result in worse outcomes than either planned, attended community births or hospital births [17, 18]. Giving birth without trained attendants intentionally (UAB) can appear a provocative choice; popular media has often painted these women as incompetent and irresponsible [19].

Although the literature suggests that planned unassisted births are on the rise [20], we do not know the percentage of unattended OOHB births that are intentionally planned, or the reasons women have for making this choice. While the statistics on planned, attended OOHB are available and positive in terms of safety, there are no statistics about the relative safety of intentionally choosing to give birth with no attendant.

There is no data to portray how demographic characteristics might influence the choice for UAB. For OOHB more broadly, researchers have found that there was a clear relationship between birth attendant and ethnicity; nurse-midwives and other midwives almost exclusively attended community births to white mothers [21]. Overall, midwives attended 43.4% of all community births, however, they attended less than 6% of community births to Black mothers; instead, physicians (52.0%) and “other” (unspecified) persons (42.3%) attended almost all (94.3%) of these births [21]. Black women are more likely to give birth out of hospital without a professional attendant than are white women; discrepancy exists between birth certificate reports and prospective study reports (Declercq, personal communication). Since physicians generally do not attend planned OOHBs in the United States, a possible explanation is that this statistic reflects births that happened quickly at home or in route to the hospital and are thus classified as being attended by a physician when they arrive at a hospital. A contributing factor to this may be the disparity in pre-term birth rates for Black women in the United States [22, 23]. Risk selection is another factor that may lead to fewer midwife-attended community births with Black women. Community-based midwives usually refer women with risk factors to hospital-based care. Some risk factors, such as hypertension and gestational diabetes, are known to occur at higher rates among Black women than among white women [22], so this could help explain a portion of this community birth stratification by skin color [21, p. 477].

Choice of provider and place of birth may also be influenced by women’s previous experiences of trauma; qualitative studies to date have shown that for survivors of trauma, birthing choices are informed at least in part by the perceived control a survivor may have over her environment and experience [24, 25], and that the choice to birth unassisted may also be in part influenced by previous unacceptable obstetric care in hospital settings [26].

More information is needed to better understand how situated racial identities and past negative experiences, trauma, and discrimination may be contributing to birth choices that trauma survivors and Black women are making. This article presents findings from in-depth interviews with women who have chosen community birth, including unattended birth, and provides evidence of decision-making regarding their birth choices. It does not shed light on the relative safety of UAB, but instead provides insight into lived experiences and social determinants of health that affect a woman’s choice of birthplace and whether or not to hire a trained birth attendant.

## Materials and methods

### Sampling method

A convenience sample of women who self-identified as survivors of trauma and/or Black and who were either interested in OOHB and/or achieved OOHB were recruited to participate in qualitative interviews. Sample recruitment began with contacting past participants to the University of Michigan STACY study. The STACY study obtained a waiver of documentation of written consent from University of Michigan IRBMED and used a phone consent process to recruit a total of 1581 women. These participants were asked about their willingness to be contacted for future participation in follow-on studies during the first phone interview.

Our study obtained separate approval from University of Michigan Health Sciences and Behavioral Sciences Institutional Review Board to expand the study beyond the STACY project. Women who had indicated they desired OOHB (whether or not they achieved OOHB) were recruited to participate in qualitative interviews. Two women were interviewed from the STACY study; both women had in fact achieved OOHB. After that, recruitment followed standard anthropological "snowball" sampling. We also distributed fliers to southeast Michigan locales frequented by childbearing women, such as community centers, social service agencies, and grocery stores. One of our first interviewees recruited via snowball sampling indicated she had given birth out of hospital unattended and provided us with information leading to an online listserv. This listserv, led by Laura Caplan Shanley, serves women interested in unattended OOHB, a phenomenon often referred to as “freebirth.” We contacted Ms. Shanley, and she distributed our flier to the listserv. This led to the recruitment of the remainder of the 16 women interviewees, who were recruited from 2009 to 2011.

After initial phone or Internet contact and screening to verify a history of trauma and/or self-identified race/ethnicity, each participant was mailed a hard copy of our IRB-approved informed consent agreement. Once the agreement was returned, interviews were scheduled in person at the participant’s home, at a coffee shop, at our office, or via phone, responsive to the participants’ choice. Although participants had the option to waive audiotaping, all 18 participants agreed to be audiotaped. Subject identifiers were stored separately from the interview data, and each participant was invited to provide a pseudonym by which to be identified. Study participants who completed interviews were compensated $40 for their time.

### Interview protocol

Interviews lasted between one to two hours in length. The interviews included open-ended questions and assessment for trauma, via the Life Stressor Checklist (LSC); the LSC assesses for 29 potential trauma exposures including behaviorally-specific inquiry into childhood and adult physical abuse and sexual abuse [27]. As the study progressed, questions related specifically to understanding unattended/unassisted OOHB were added. Main areas of interest covered during the interviews were how and why women chose OOHB, whether their past experiences were contributing factors, and if so, how. For example:

1. Did you “choose” to give birth there, or did it “just happen”?
2. Why did you make that choice?
3. Is there anything from your past that influenced your decision of where to have your baby?
4. Many women have fears about having their first baby—did you? Did your fears influence your decision?
5. What informed your decision to have unassisted childbirth this time around?

### Data analysis

Our study sought basic information about the decision-making process concerning OOHB and UAB. We employed a *grounded theory* approach [28] with the hope that by exploring the participants’ shared lived experiences we might uncover insight into an initial theory regarding OOHB/UAB decision-making. We followed a sequenced approach [29] that included beginning with a broad focal area, delaying literature review until later in our research, conducting simultaneous data collection and initial analysis, conducting constant comparison of data, codes, and memos, theoretical sensitivity to emergent themes that correspond to broader literature findings, and theoretical sampling also respondent to saturation of categories and theory development (in this case, shifting from looking exclusively at OOHB and including UAB [29]. Because such an approach assumes no a priori judgment about findings, it involves a discursive process of data collection, theory generating, and memo writing to reach conclusions [30]. Basic procedures included a process of *open coding*, whereby transcripts of interview or other textual data were coded into major categories, *axial* or selective coding (also called “focus coding”) to identify core phenomena of central focus, *memoing* or writing down ideas about theory emerging from the data, and conceptual modeling/theory generation [30, 31]. The researchers independently open- and focus-coded the interview narratives, and subsequently discussed any discrepant interpretations until consensus was achieved prior to theory generating.

## Results

Table 1 provides information about the self-identified race/ethnicity, number of live births, place of birth, and history of lifetime traumatic exposures of the interviewees.

**Table 1:** Information about participants: ethnicity, number of live births, birth places, and lifetime trauma exposure

Pseudonym	Ethnicity	Total # of live births	Hospital births	Planned Out of hospital births	Un-assisted births	Lifetime trauma exposures
Natalie	White	4		2	2	2
Renee	Black	5	3		2	18
Ophelia	Black	4	2	1	1	15
Angelina	Black	2	1		1	9
Maggie	White	2			2	5
Julianna	White	3		1	2	9
Sally	Black	7	4		3	15
Jay	Black	2	1	1		20
Cara	White	1		1		17
Sophia	White	1		1		12
Dana	Black	5	4	1		9
Nicole	Latinx	2	1	1		9
Rhiannon	White	1		1		5
Nightsong	White	2	1 cesarean	1 (VBAC)		11
Mariah	Black, mixed-race	1		1		7
Gracie	White	2	1	1		14
Greta	White	1		1		3
Garden Lady	White	1		1		10

### An emergent model

**Fig. 1:**
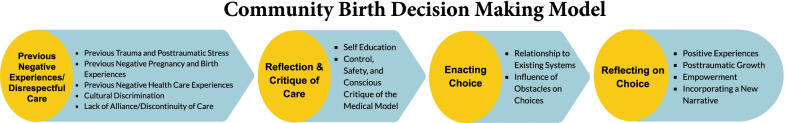
Model of decision making

Four key themes coalesced into a simple model of a trajectory from negative past experiences to a period of reflection on these experiences, to enacting different choices, and ultimately reflecting on these choices (Fig. 1). The first key theme was that of previous negative experiences with pregnancy, birth, and other medical healthcare. This included connections between previous trauma/posttraumatic stress and current health seeking, previous negative pregnancy and birth experiences, previous negative healthcare experiences, experiences of cultural discrimination and racism, a lack of alliance with healthcare providers, and a sense of discontinuity of care.

The second emergent theme was about reflecting on and critiquing previous care received or extant models of care. This theme related to the ways in which women sought to educate themselves about pregnancy and birth, expressions of the need for control and safety, and conscious critiques of the medical model of care.

The third key emergent theme related to the ways in which women enacted an out-of-hospital birth plan, including unassisted birth. This included introspection about relationships to existing systems of care, and the influence of obstacles on choices regarding care. The fourth key theme related to women’s reflections on their birth choices. These included stories of positive birth experiences, instances of posttraumatic growth and empowerment, and the incorporation of a new narrative about their choices and experiences.

### Previous negative experiences/disrespectful care

All the women in this study discussed previous negative experiences related to trauma and/or disrespectful care. Women shared about previous life trauma and resulting posttraumatic stress. These previous life traumas included healthcare-related trauma that occurred during previous pregnancies, labors, and other healthcare experiences, as well as discrimination and cultural trauma.

#### Previous trauma and posttraumatic stress

Women in this study self-identified as women of color (eight out of eighteen) and/or women who had experienced sexual abuse (eleven out of eighteen). Women for the most part did not choose to elucidate their history of abuse, although they did speculate about ways in which having a history that includes sexual abuse and symptoms of posttraumatic stress might affect their birth care:

I was afraid that with my history, that if she said the wrong word at a very vulnerable time during the birth that I would’ve had a flashback or something. (Nightsong)

Ophelia related what sharing her history of abuse with her midwife looked like, and how that related to the concept of control:

I think the sexual abuse that I’d experienced as a child, I needed, part of me, because my midwife asked me, “What do you want us to do?” And I said, “I need to do it myself. That’s how I’m going to feel in control.”

For Sally, her past trauma contributed to a sense of wanting a birth experience that was just between her and her husband (unattended):

With that first pregnancy, it’s just kind of a lot. I think too maybe, when it comes down to maybe having traumatic experiences, that maybe would weigh on my decision to have it just be my husband and myself. (Sally)

#### Previous negative pregnancy and birth experiences

Twelve out of eighteen of the women in this study related stories about previous negative reproductive healthcare experiences, some of which were endorsed as traumas by the participant, and reflected on how those experiences shaped their thinking about subsequent birth experiences. Renee and Julianna described this in relation to vaginal examinations during prenatal care:

At the OB, she would examine me and then she would ask her students in there, and there’d be like 12 of them and I’d just be frozen. And I just feel like, nobody should...I understand that people need to get their experience, but why do there have to be 12 of you? You all don’t have to be in my vagina. (Renee)

It left me feeling like I had been raped, really. With a sense of like, “I had just been taken advantage of.” Even though he was perfectly professional. I’m sure, you know, it was his business. It left me with a feeling of “I’d just been molested” feeling. I remember what it felt like to feel like that, and that’s what it felt like. So I never went back to him. (Julianna)

Ophelia described a previous birth experience, “My first one, they held my hands down and wouldn’t let me touch her. It was a traumatic birth in a lot of ways.” Jay also described a previous birth experience:

They held my legs together for 25 minutes while she was completely coming out of me. It damaged part of her brain. After he (the doctor) got there and just cut me, and then after she was born, he started stitching me up without any Novacaine and I said, ‘You’re going to have to stop because I can feel all of this.’ And I really wanted to have a home birth after that! (Jay)

#### Previous negative healthcare experiences

Several women related instances of negative experiences interfacing with healthcare providers that did not involve birth itself. These included a car accident, terminal cancer care of a child, emergency room care for a multiple sclerosis relapse, and other events. Garden Lady recounted her story of being in a motor vehicle accident:

I'd been in a car accident... [I had] whiplash, and it was really not fun, and I've got this calcified knot in my neck. And so I went in and I was like, “You know, this is really bad. It really hurts. I'd really like to deal with this somehow.” And she [a doctor] reached back, “Oh no, you're fine.”... I was just so stunned. Wow, it doesn't exist. Yeah, I was just like, ok, um. I need to find another doctor now.

For Sally, a previous negative experience with the medical community was a four-year period spent interfacing with hospital personnel while her daughter was dying from brain cancer. She recounted that she was

[…] kind of on hospital overload, I think, and just overwhelmed with watching her go through cancer and the different medications and just different things and different practices at the hospital that we just weren’t pleased with and the way things went.

In addition to dealing with medications and procedures, Sally and her family also experienced financial hardship which led to the experience of feeling discriminated against based on socio-economic status:

It crippled us, emotionally, financially, everything. We ended up having to go on public services, having to get MediCal, different stuff. When we got on MediCal, we got treated like we were on MediCal. Let’s just put it like that. Literally. We felt like they didn’t care. We felt like they had no sense of ownership for our daughter’s care or her outcome…(Sally)

#### Cultural discrimination

In addition to medical care that felt disrespectful or even traumatic, many participants related that they felt discriminated against because of their young age, skin color, weight, or socioeconomic status, or intersecting aspects of their identity. Here are two stories from Renee and Angelina, both of whom are Black, that exemplify these experiences, though this was a pervasive theme across accounts:

I wear head wraps and one day my head wrap came off and she said, “Oh, you look like the Bride of Frankenstein.” I have pretty nappy hair and I don’t appreciate the comments about my hair. …and not even that she was intending to be disrespectful, because she thought it was a joke, but she couldn’t understand the connotation of what that means because she wouldn’t understand my background. (Renee)

I had a horrible, horrible MS [multiple sclerosis] attack. My doctor was out of the state. So I went to the doctor that was on call for her. I was going blind, I couldn’t feel anything from the waist down, I was incontinent. He (the doctor) told me, “It’s obviously a back injury because you’re not a small woman.” And I said, “Why am I going blind? Is fat growing over my eyeballs? Why am I going blind?” (Angelina)

#### Lack of alliance/discontinuity of care

Some women reported experiencing a lack of alliance with their care provider, or instances of discontinuity of care, regardless of previous negative healthcare interactions or discriminatory or insensitive care experiences. As Dana said, these women described feeling like “the person in the patient is forgotten.”

[…] the best prenatal care that I received to date, there’s always been just a disconnect between myself and the care providers. Sometimes it’s like […] yeah, a disconnect between myself and the care providers. (Renee)

She was a nice enough lady, and she came recommended from a friend of mine. I just felt like, she just wasn’t connecting – we weren’t connecting. (Mariah)

I knew that it would be the luck of the draw as to who would actually be there and it might not necessarily be that one of seven, or nine, that we’d really bonded with. So that was my whole worry. (Natalie)

And sort of feeling like, “Why are we coming in here?” We could just have robo-midwives, you just walk in and step on a scale and pee in a cup and just leave. (Garden Lady)

### Reflection and critique of care

Those previous negative experiences (birth or non-birth-related) led these women to seek out new information and engage in a process of discernment about what was right for them for future birthing experiences. This education and discernment process also strengthened their critiques of the medical model. Out of this process, women made the decision to give birth outside of a hospital either with a homebirth midwife or unassisted (by choice or because of obstacles to hiring a midwife).

#### Self-education

Women engaged in an extensive process of educating themselves about the birth process and their range of options. These included engagement with popular media, online sources, and print materials.

*Internet interactions* Many women spontaneously criticized mainstream books and media representations of birthing women. They perceived that the media creates a cultural message that birth is a painful emergency. Nightsong describes the way she educated herself before her first birth:

I was, unfortunately like so many women out there today are, pretty clueless about how birth really works. I thought I was informed, but I wasn’t in the least bit informed. I went the hospital birthing class and the hospital breastfeeding class - neither one of which were really helpful. I thought they were. Looking back - not in the least. (Nightsong)

Not surprisingly, women turned to the Internet, seeking educational and inspirational stories, articles, and videos. They also turned to online discussion forums, such as the one associated with Laura Shanley, author of *Unassisted Childbirth,* published in 2016 [32], where stories about UAB are posted, questions are answered, and extensive “how-to” knowledge is shared.

Dana describes how interactions in an online parenting forum changed her birth trajectory. She had three vaginal births in hospitals and then gave birth to her fourth child, who had spina bifida, by cesarean. She intended to give birth to her fifth child vaginally in a hospital. Around 36 weeks of pregnancy, her doctor ordered an ultrasound. The ultrasound technician did not think there was anything unusual about her baby’s size. Dana describes the interaction with her obstetrician after that:

“So everything looks good?” She said, “Yeah, it looks like you have a big baby with a BIIIIG head!” And she really just emphasized that and at that point, I was still like, “This is just weird. Why is she acting so weird?” I happen to be a member of a natural parenting forum...Someone had said to me, “Had you ever given thought to the fact that she might trying to convince you [that] you had a big baby, to try to convince you that the c-section is a better route to go?” And it was at that time that I thought, “You know what – that makes sense.” I put all the puzzle pieces together and I felt like that was right on. It was at that moment in time that I lost complete confidence in her caring for me...I thought, “This is not what I’m doing. I’m not having a c-section.” I’m not having a c-section. She’s not pushing me into having a c-section.

*Reading* Scholarly reading also played significant roles in the self-education process for women:

I just read a lot! (…) I was reading studies, reading journals, like when I could get *Midwifery Today*. If I go to my library then I have access to J-Stor. I read old scholarly works. Different things like that really influenced the perception that I had of birth, and the perception that I had on the role of the woman. That all influenced how I felt about my birth. (Renee)

I feel like I read! Gosh! I had a reading list, I remember. It had like a dozen books on it. I read them all. I read *The Thinking Woman’s Guide to Giving Birth*. I think that was probably a pivotal point. And so my mistrust for the whole process around mistreating women in pregnancy and birth began from my personal experience there and was reinforced through all my reading and research. That decision [to give birth outside of a hospital] was made after doing a lot of reading and research. (Natalie)

Other experiences also contributed to these women’s growing sense that the medical model was not right for them. For example, Jay worked as a doula and the experience of accompanying women during hospital births made her realize that achieving an unmedicated birth with few interventions was quite difficult in the hospital setting. Instead of being able to concentrate on contractions and physical sensations, Jay says that, “You just have to be on your toes and really working hard to get the birth plan that you want.” Rhiannan related the story of witnessing a friend give birth by herself because labor was too fast to get to the hospital. And Sally began to pursue a degree in Naturopathy.

*Alternative medicine *Twelve out of eighteen participants expressed a general preference for alternative or natural medicines and practices. These included preferences for natural and organic foods, herbal medicines, and vitamin supplements. Participants also shared that they used alternative healing methods like massage therapy or chiropractic care.

#### Control, safety, and conscious critique of the medical model

Previous experiences and self-education existed in a synergistic relationship with the emergence of these women’s critiques of the medical model. As women critiqued the medical model, they were often able to explain to themselves why previous experiences had felt so negative. Much of their critique focused on the issue of “safety” and feeling *unsafe* in the care of medical professionals or in the hospital environment. Every interviewee made comments that critiqued the dominant model of care and many explicitly connected “feeling safe” with making one’s own choices (“control”) about birth. For example:

[…] it bothers me a lot, I’m actually in school to be a nurse right now, it bothers me so much to know that there’s so many women that could have a birth that they may want more than another and they’re not getting it because they’re not educated – they don’t know they have a choice. (Dana)

I feel very strongly that women need to give birth where they feel safest. Or where they feel like they can relax the best. So if that's the hospital, then go to the hospital. I think that probably the most important thing is to figure out where you feel safe...I think that the best thing that I learned from this is that my intuition and my instinct is good. I know things. I'm not stupid because I don't have a medical degree. I can do this just fine. And I can learn things and...I don't always need some expert to tell me what to do. (Greta)

All the people just coming in. All of the sudden there’s all these people coming in and out and in and out and in and out. And that’s not very conducive to feeling safe and relaxed. (Gracie)

The only real fear that I had was giving birth in a hospital. Giving birth itself? No. Giving birth in a hospital? I was terrified of that. (Rhiannan)

Critiques of the medical model, coupled with a desire for more control over their own experiences, led these women to decide they wanted something different from the mainstream biomedical approach to childbirth.

### Enacting choice

#### Relationship to the existing systems: in-system, in-between systems, out-of-system

Some women came out of this period of research and discernment hoping to give birth in a hospital but with more control. Others knew, based on their research and critique of previous care, that they wanted to give birth outside the hospital-based system. A few knew that they preferred an unassisted birth. Women had to balance their own desires with those of their partner and, occasionally, extended family members. Although some were sure they wanted an unassisted birth, others found themselves betwixt and between systems, with one foot in the hospital system and another outside. As they attempted to enact their choices, some ran into obstacles such as financial barriers, lack of available midwives in their area, or interactions with care providers that gave them pause.

Maggie is an example of someone who knew that she did not want a hospital birth after a negative hospital miscarriage experience:

[…] after the miscarriage, it was totally solidified. There was no way you could pay me enough money, or compel me forcibly enough, to have a baby in a hospital.

However, she did not immediately know what she did want:

Well when we found out that we were pregnant with K--, I just kept going over in my head, I can’t possibly go back to these midwives who were the only midwives in the area. There’s no one else who would deliver at home. I refuse to deliver in the hospital and so at that point, what we’re going to do is just be pregnant for nine months and see if something turns up. What turned up was Laura Shanley’s book. I was like, “Oh, look at that. People aren’t just crazy and it happens.” So that was then how it became solidified. (Maggie)

#### Influence of obstacles on choices

Sally and Angelina also knew that they did not want to give birth in a hospital. They first attempted to hire homebirth midwives, but finally gave birth unassisted:

Ultimately with her [Sally’s daughter] passing away that year and for us to really want it to be like a very close family experience for the birth, and me wanting to have my other children involved and things like that, things that I just knew they weren’t going to do at the hospital period, made me want to have a homebirth. (Sally)

Obstacles to a homebirth midwife-attended birth led Sally to choose an unassisted birth, as she explains here:

We were going to have a midwife. There’s really only one that was available in my area and she’s like almost two hours away. For the price, because I was already seeing my OB [obstetrician] for my regular visits, and what we would have had to pay for her to just come and strictly do the delivery only, and we didn’t mesh well with her personality-wise that well either, we just decided to go ahead and try the unassisted home birth.

Angelina worried that she would not achieve the birth she wanted with the homebirth midwife she originally hired:

[…] my daughter’s birth really tainted me because I didn’t want anyone to give me permission to do something or not do something. Period. I wanted to be completely in control. And I didn’t want someone saying, “No, I have to put my hand in your vagina first and make sure that you can push or lay down to push.”

I started feeling really strongly that I wanted the first person that touched our baby to be my husband. Period. I didn’t want it to be a midwife, I didn’t want it to be someone else. I asked the midwife…how she felt about that and she said, “Well, I have to be there and I have to be checking things during your labor. I have to be there and I might have to catch the baby. You don’t know what will happen.” It just felt wrong to me. It just felt really important to me to have my husband catch the baby. So that’s when we decided not to do homebirth midwives... when we left, we sat and looked at each other across the table at lunch. And I said, “Unassisted?” and he said, “Yeah, unassisted.” And that was it!

Renee, like one other woman in our study, was unable to find a local homebirth midwife to attend her. She says:

It’s hard to find a homebirth midwife, and then the lay midwife that I did find, her philosophy was in line with mine. She was like, “I’m on hiatus from doing births, but you can have an unassisted birth and I’ll help you. I’ll give you information.”

Gracie told us that she had bowed to her partner’s wishes for their first child’s birth, which she described as, “suction, forceps, suction, forceps, finally pulled, yanked out with forceps,” because he

[…] at that point, was not totally supportive of the idea of not having a hospital birth anyways. I mean, even if we could have (financially). So the fear of what might happen, all of the complications and whatever.

But, going into her second birth experience, she says,

I think I didn’t care what people thought the second time. The first time I was weighing in, thinking like, “Maybe they’re right. Maybe it is really too dangerous. And maybe it is a crazy idea.” My family thought it was a crazy idea. [The second time], I knew that it was going to be different. That it had to be different. That this was my opportunity, and it was going to be different.

Whether women ultimately made the choice to birth within the hospital system, or to go outside the system, considerable discernment was evident related to decision-making and choosing birthplace and providers (or lack thereof). Renee is conscious of this process and its implications:

As long as being an adult means making decisions and then also dealing with the consequences of whatever decision that you make and as long as you’re making an informed decision and you have weighed the consequences then you know, you have to do what you feel is best for you.

### Reflecting on choice

Women overwhelmingly described their community births, including their unassisted births, as positive experiences that generated posttraumatic growth, healing, and feelings of empowerment. This contrasted sharply with the tone and content of stories about mainstream hospital births that they had seen or experienced. Many women spontaneously described these births in spiritual terms.

#### Positive experiences

The examples of Jay, Ophelia, Gracie and Sally show how positive these stories were:

(I) had such a great time with it, I thought, “Oh, let’s do this again.” (Jay)

[…] the birth itself was incredible. It really was. I got to catch my own baby in the pool at home. (Ophelia)

I had no idea I was having the baby. If you had told me that I was giving birth at that moment, I would never had guessed. I mean, I felt funny. And so three pushes and the baby came out. And my instant reaction was, “Oh my gosh! I have to do that again!” I mean that was such a different experience. I was just like that was awesome and the very best thing that’s ever happened and I have to do it again. I’m not going to, but that was my feeling. (Gracie)

This… birth, it’s just like we knew what to do. We were, front to back, we knew exactly what to do and how to do it, and it was just perfect. It was literally just perfect. (Sally)

*Posttraumatic growth *Not only were these birth experiences positive, but women also described them as healing (in relation to their previous traumas). They saw that they had new reactions, feelings, and thoughts about previous sexual abuse and traumatic births:

I knew from the moment my husband and I first had sex after the baby was born, the birth itself healed me from sexual abuse. It was amazing! It was clear as day for me. Of course, I love my (first child) beyond words, but the birth itself, how traumatic that process was -- it almost didn’t matter because the second birth healed me from the first birth. Beyond that, the second birth healed me from my sexual abuse. I had always had problems during sexual intercourse with just being able to relax, and it always took a long time for my husband to be able penetrate and that kind of thing, and from the very first time we made love after my son’s birth, there was no problems at all and there hasn’t been since. (Nicole)

I didn’t know that I had fears to confront for real. After I gave birth, especially like this last time, I knew, this is strange, but I knew that I had lost fears. I felt absolutely fearless about my ability to get my grown woman on. To be a grown woman and do what I have to do. (Renee)

*Empowerment *In addition to noting these instances of what might be described as posttraumatic growth, women attributed new feelings of empowerment to their birth experiences:

The empowerment through giving birth on my own terms has definitely spilled over to all aspects of my life, for sure. I am clearly a different person. Through that, and that I was a few years ago after giving birth and I would not be that same person in the positive aspects if I had given birth [differently]. (Natalie).

I would recommend her to, if she was a spiritual person to see birth as a spiritual journey and to see it as an evolution of herself as a woman. That labor, thinking about labor and delivery, not that labor and delivery that it’s not only a chance for you to meet your child, but it’s a chance for you to get to know a facet of yourself that you wouldn’t otherwise know. (Renee)

*Incorporating a new narrative *Similar to the sentiment expressed by Natalie about feeling like a different person, others also expressed ways they were incorporating a new empowered narrative about themselves based on their experiences giving birth:

Since I’ve given birth, when I think of my body, I think whole, capable -- capable is a good word -- and strong. How empowering birth is. Homebirth is unmedicated and you’re in a loving environment, surrounded by encouraging people. You feel so powerful and so, kind of like an Earth Goddess. It’s something that can’t be replicated. (Rhiannan)

I just always heard before I birthed the first time that it was this painful, awful thing. It was something you have to go through in order to have a child and it was terrible and tragic and it was Eve’s sin that caused all of this. I remember as a child wanting children – I always wanted to be a mom but I was always terrified of actually giving birth. Now of course, that’s another great learning that came from this – that it doesn’t have to be that way. And it’s only that way if you let it be that way. Like I said, in fact, it can be a completely empowering, wonderful, best experience of your life kind of thing. And it truly was. The day I gave birth to my son was the best day of my life. (Nicole)

## Discussion

When a woman in the United States suggests she is considering a community birth with a trained midwife, she is likely to encounter strong pushback from family, friends, and colleagues based on commonly held beliefs about “safety” and “risk.” Women are told they are “selfish” and “naïve” (https://www.theguardian.com/commentisfree/2011/nov/27/barbara-ellen-birth-marilyn-monroe). Even more attacked are women who plan unattended births. The story of “unattended births” has often been told through a “monster mother” lens [19, 33], and is tinged with judgment and moralizing, as authors wonder, “How could she put her baby so at risk?” This paper, following Greenfield, Jomeen, and Glover, 2019 [4], invites new interpretations. It codifies a process of decision making that may seem intuitive to many birth professionals, but which has, to our knowledge, never before been modeled. The stories reveal previous troubling medical care patterns, which demand closer attention.

In 2010, Bowser and Hill [34] identified seven categories of disrespectful and abusive care. One aspect of disrespect that is applicable to the women in our study is the abrogation of women’s right to make decisions about their own bodies in labor, delivery, and postpartum. Since then, at the global level, the connection between disrespectful care, undesirable outcomes, and future avoidance of care has been well-studied (e.g., [35], page S50).

The theoretical lens of “disrespectful care” shines a light on the unequal power dynamic between medical professional and birthing woman. As Metzl, Kirkland, and Kirkland wrote in 2010 [36], “health is a concept, a norm, and a set of bodily practices whose ideological work is often rendered invisible by the assumption that it is a monolithic, universal good” (p. 9). They point out that when people act against hegemonic, biomedical definitions of health (for example, by smoking) they are judged to be bad people. However, they also note that when doctors, patients, and policymakers talk about health, “they are not all talking about the same thing” (p. 9). In particular, when birthing mothers who have experienced trauma, abuse, or discrimination refer to “safety,” they probably have very different concepts in mind than their medical providers. Medical providers may also be unaware that Black women report experiencing racial discrimination and unfair treatment in healthcare (and other settings) during pregnancy, which places them at increased risk for prenatal depression [37].

Though many out-of-hospital midwives and mothers may have, themselves, experienced strong cultural approbation for their non-mainstream birth choices, they may be skeptical about the unassisted or “freebirth” movement. In the dominant birth narrative, medical emergencies that can occur during labor, birth, or the immediate postpartum period are so risky that the only rational choice is to give birth with a trained medical professional. Women who reject such help, in this interpretation, may be seen as selfish, uneducated, and/or making uninformed or unwise choices.

Our research challenges this. In its place, we offer an understanding of the pathway to unassisted birth, grounded in women’s own stories, that implicates the biomedical model of American hospitals (and, sometimes, homebirth practice). The women in this study took their decision to give birth outside of a hospital and/or unassisted extremely seriously and were well-aware of how counter-cultural their choices were. Their descriptions of research and self-education matches the findings of Jackson, Dahlen, and Schmied in 2010 [38], who found that “in pursuing the best for themselves and their babies, women who birth outside the system spent a lot of time and energy considering the risks and weighing these up” (p. 566). Another study of women who had previously experienced traumatic hospital births also found that these women, in subsequent pregnancies, spent a lot of time gathering information [4].

Contrary to mainstream depiction, these women describe themselves as educated and well-informed. In fact, they may see women who follow obstetric advice blindly, without doing their own research, as uneducated. Researchers in Canada found, similarly, that women who made alternative birth choices (including hiring a doula in a hospital or giving birth outside of a hospital) felt they were judged as “deviant,” but utilized information management techniques to “present themselves as responsible, competent mothers” [39].

For all of the participants, previous negative experiences played a pivotal role in their decision to give birth outside a hospital, with or without assistance. Instead of feeling safe inside a hospital with medical caregivers, these women felt unsafe and unsupported. This was true at the physical and the emotional level. Physically, women in this study were uniformly clear that they wanted physiologic births, which is difficult to achieve in the United States. Generally, they wanted to maintain control of who touched them, when they were touched, and in what ways. Emotionally, women desired to give birth in conflict-free, supportive environments in which their bond with their baby was respected. Fears that medical interventions would happen without consent (or through coercion), that they would be separated from their babies, or that caregivers would be “cold” or “clinical” (all elements identified in the “disrespectful care” literature) underline these women’s sense that hospital birth is “riskier” than caregivers think it is. Recently, a large quantitative study of 2700 birthing women in the US found that one in six women reported experiencing caregiver maltreatment, including verbal abuse, stigma, and discrimination [40]. It also included being ignored when requesting help.

Asking for help and being ignored stands in bas relief to another aspect of maternity care, which is choice concerning the provision of medical interventions, which are ubiquitous and may be hard to refuse. Recent research found that women who declined intervention, especially women of color, were more likely to experience discrimination [41]. Conversely, provision of care for pain may be denied to women of color based on racial bias [42]. These experiences of discrimination and racism in medical settings are common and likely influence healthcare seeking in general.

Women in our study began to see their desire to avoid unnecessary medical interventions and to be in charge of their own bodies (for example, how many people viewed their naked body and the occurrence of vaginal exams) as sites of struggle. As Natalie says, “Yeah, it was a leaning-towards-natural-birthing-in-the-hospital initially. Which, as we began to explore what the obstacles were for that and what challenges we’d be facing, we realized we were arming ourselves for battle.”

Avoiding unwanted interventions seemed nearly impossible in the hospital setting. When Mariah says, “I just really didn’t want a male doctor there and I didn’t want a c-section and that was about it” to describe her “freebirth” choice, she is implying that she would not have full control over those choices in a hospital. Women who had previously given birth were especially aware that it can be hard to advocate for oneself in labor. These themes were consistent with the findings evident in a 2016 review of fifteen articles about birth outside of medical systems [43], in which women describe viewing the hospital as “a dangerous place” and “feeling that true autonomous choice is only possible at home” (p. 61).

As we analyzed these stories, the pattern of negative experiences, subsequent research, and out-of-the-mainstream decision-making, became clear. Their jubilance and sense of triumph at accomplishing birth on their own terms was palpable. These positive experiences led to posttraumatic growth and enhanced self-confidence, which other researchers have also noted (e.g., [44]).

Limitations of this study include the fact that this model is not the only model that could explain community or unassisted birth choices. Women who have not experienced previous negative interactions with medical care providers, who have not experienced discrimination, or who have not been exposed to trauma may also choose community or unassisted birth, but for different reasons. In addition, in these stories, all the mothers and babies were free from medical complication. Their reflections might have sounded quite different if there had been a serious complication. Another limitation is that this is a small sample of women who were motivated to share their intentional and positive experiences of unassisted birth.

## Conclusion

Our qualitative research showed that previous trauma and experiences of discrimination were influential factors for women in their choice of birthplace setting and choice of provider. Given the ubiquity of racism, gender discrimination and sexual abuse in modern society, and reports of disrespectful care in medical settings, this research should underscore the necessity of trauma-informed, antiracist, culturally congruent, and respectful medical care at all levels, especially in the reproductive health context. Learning from mothers who have rejected the healthcare system as a safe system in which to give birth can help all of us reflect on and improve care.

This study reframes the social discourse around what makes a “good mother.” We think opening the definition of “good mother” to include women who have chosen UAB allows a focus on women as autonomous and logical decision makers who are capable of making nuanced decisions, rather than passive “patients” who must listen to medical advice. We invite medical care providers and policy makers to consider bodily autonomy, physical and emotional safety, anti-racism, and independence as equally important factors in high-quality parturient care.

## Data Availability

The datasets analyzed during the current study are not publicly available due to inclusion of potentially identifying characteristics but are available from the corresponding author on reasonable request.

## Abbreviations

OOHB: Out of hospital birth
UAB: Unassisted birth

## References

[1] MacDorman MF, Declercq E (2019). Trends and state variations in out-of-hospital births in the United States, 2004–2017. Birth.

[2] Cheyney M, Bovbjerg M, Everson C, Gordon W, Hannibal D, Vedam S (2014). Outcomes of care for 16,924 planned home births in the United States: the Midwives Alliance of North America Statistics Project, 2004 to 2009. J Midwifery Womens Health.

[3] MacDorman MF, Menacker F, Declercq E (2010). Trends and characteristics of home and other out-of-hospital births in the United States, 1990–2006. Natl Vital Stat Rep.

[4] Greenfield M, Jomeen J, Glover L (2019). “It can’t be like last time”—choices made in early pregnancy by women who have previously experienced a traumatic birth. Front Psychol.

[5] Boucher D, Bennett C, McFarlin B, Freeze R (2009). Staying home to give birth: why women in the United States choose home birth. J Midwifery Womens Health.

[6] Wiegers TA, Keirse MJ, van der Zee J, Berghs GA (1996). Outcome of planned home and planned hospital births in low risk pregnancies: prospective study in midwifery practices in the Netherlands. BMJ.

[7] Hutton EK, Reitsma AH, Kaufman K (2009). Outcomes associated with planned home and planned hospital births in low-risk women attended by midwives in Ontario, Canada, 2003–2006: a retrospective cohort study. Birth.

[8] Hadjigeorgiou E, Kouta C, Papastavrou E, Papadopoulos I, Mårtensson LB (2012). Women's perceptions of their right to choose the place of childbirth: an integrative review. Midwifery.

[9] Lederman RP, Lederman E, Work BA, McCann DS (1979). Relationship of psychological factors in pregnancy to progress in labor. Nursing Res.

[10] Epstein CM, Houfek J, Rice MJ, Weiss SJ (2021). Integrative review of early life adversity and cortisol regulation in pregnancy. J Obstet Gynecol Neonatal Nurs.

[11] Seng JS, Low LK, Ben-Ami D, Liberzon I (2005). Cortisol level and perinatal outcome in pregnant women with posttraumatic stress disorder: a pilot study. J Midwifery Womens Health.

[12] Seng JS, Li Y, Yang JJ, King AP, Low LM, Sperlich M, Rowe H, Lee H, Muzik M, Ford JD, Liberzon I (2018). Gestational and postnatal cortisol profiles of women with posttraumatic stress disorder and the dissociative subtype. J Obstet Gynecol Neonatal Nurs.

[13] Seng JS, Kohn-Wood LP, McPherson MD, Sperlich M (2011). Disparity in posttraumatic stress disorder diagnosis among African American pregnant women. Arch Womens Ment Health.

[14] Sanjuan PM, Fokas K, Tonigan JS, Henry MC, Christian K, Rodriguez A, Larsen J, Yonke N, Leeman L (2021). Prenatal maternal posttraumatic stress disorder as a risk factor for adverse birth weight and gestational age outcomes: a systematic review and meta-analysis. J Affect Disord.

[15] Zielinski R, Ackerson K, Low LK (2015). Planned home birth: benefits, risks, and opportunities. Int J Womens Health.

[16] Cook D, Avery M, Frisvold M (2014). Formulating evidence-based guidelines for certified nurse-midwives and certified midwives attending home births. J Midwifery Womens Health.

[17] Burnett CA, Jones JA, Rooks J, Chen CH, Tyler CW, Miller CA (1980). Home delivery and neonatal mortality in North Carolina. JAMA.

[18] Schramm WF, Barnes DE, Bakewell JM (1987). Neonatal mortality in Missouri home births, 1978–84. Am J Public Health.

[19] Ginsburg FD, Tsing AL (2001). Uncertain terms: negotiating gender in American culture.

[20] Hickman A (2009). Born (not so) free: legal limits on the practice of unassisted childbirth or freebirthing in the United States. Minn L Rev.

[21] Declercq ER, Paine LL, Winter MR (1995). Home birth in the United States, 1989–1992: a longitudinal descriptive report of national birth certificate data. J Nurse-Midwifery.

[22] Rosenberg TJ, Garbers S, Lipkind H, Chiasson MA (2005). Maternal obesity and diabetes as risk factors for adverse pregnancy outcomes: differences among 4 racial/ethnic groups. Am J Public Health.

[23] Alhusen JL, Bower KM, Epstein E, Sharps P (2016). Racial discrimination and adverse birth outcomes: an integrative review. J Midwifery Womens Health.

[24] Montgomery E (2013). Feeling safe: a metasynthesis of the maternity care needs of women who were sexually abused in childhood. Birth.

[25] Montgomery E, Pope C, Rogers J (2015). The re-enactment of childhood sexual abuse in maternity care: a qualitative study. BMC Pregnancy Childbirth.

[26] Diamond-Brown LA. Women’s motivations for “choosing” unassisted childbirth: A compromise of ideals and structural barriers. In Reproduction, Health, and Medicine 2019 Nov 22. Emerald Publishing Limited.

[27] Wolfe J, Kimerling R, Wilson JP, Keane TM (1997). Gender issues in the assessment of posttraumatic stress disorder. Assessing psychological trauma and PTSD.

[28] Glaser BG, Strauss AL, Strutzel E (1968). The discovery of grounded theory; strategies for qualitative research. Nurs Res.

[29] Qureshi HA, Ünlü Z (2020). Beyond the paradigm conflicts: a four-step coding instrument for grounded theory. Int J Qual.

[30] Padgett DK. Qualitative methods in social work research. Sage publications; 2016 May 25.

[31] Creswell JW (2007). Qualitative inquiry and research design: choosing among five approaches.

[32] Shanley LK. Unassisted childbirth. ABC-CLIO; 2012 Feb 22.

[33] McDonald K, Amir LH, Davey MA (2011). Maternal bodies and medicines: a commentary on risk and decision-making of pregnant and breastfeeding women and health professionals. BMC Public Health.

[34] Bowser D, Hill K (2010). Exploring evidence for disrespect and abuse in facility-based childbirth.

[35] Miller S, Lalonde A (2015). The global epidemic of abuse and disrespect during childbirth: history, evidence, interventions, and FIGO's mother–baby friendly birthing facilities initiative. Int J Gynecol Obstet.

[36] Metzl J, Kirkland A, Kirkland AR (2010). Against health: how health became the new morality.

[37] Ertel KA, James-Todd T, Kleinman K, Krieger N, Gillman M, Wright R, Rich-Edwards J (2012). Racial discrimination, response to unfair treatment, and depressive symptoms among pregnant black and African American women in the United States. Ann Epidemiol.

[38] Jackson M, Dahlen H, Schmied V (2012). Birthing outside the system: perceptions of risk amongst Australian women who have freebirths and high risk homebirths. Midwifery.

[39] Bayly M, Downe P (2018). "Most often people would tell me I was crazy": defending against deviance ascribed to alternative birth choices. MIRCI.

[40] Vedam S, Stoll K, Taiwo TK, Rubashkin N, Cheyney M, Strauss N, McLemore M, Cadena M, Nethery E, Rushton E, Schummers L (2019). The giving voice to mothers study: inequity and mistreatment during pregnancy and childbirth in the United States. Reprod Health.

[41] Attanasio LB, Hardeman RR (2019). Declined care and discrimination during the childbirth hospitalization. Soc Sci Med.

[42] Hoffman KM, Trawalter S, Axt JR, Oliver MN (2016). Racial bias in pain assessment and treatment recommendations, and false beliefs about biological differences between blacks and whites. Proc Natl Acad Sci USA.

[43] Holten L, de Miranda E (2016). Women’s motivations for having unassisted childbirth or high-risk homebirth: an exploration of the literature on ‘birthing outside the system’. Midwifery.

[44] Feeley C (2019). Freebirthing: a case for using interpretative hermeneutic phenomenology in midwifery research for knowledge generation, dissemination and impact. J Res Nurs.

